# Autoimmune disease: genetic susceptibility, environmental triggers, and immune dysregulation. Where can we develop therapies?

**DOI:** 10.3389/fimmu.2025.1626082

**Published:** 2025-08-07

**Authors:** Manoj Kumar, Linda Yip, Fangyuan Wang, Saci-Elodie Marty, C. Garrison Fathman

**Affiliations:** ^1^ Department of Medicine, School of Medicine, Stanford University, Palo Alto, CA, United States; ^2^ Department of Radiology, School of Medicine, Stanford University, Palo Alto, CA, United States

**Keywords:** Autoimmune disease, genetic susceptibility, environmental triggers, regulatory T cells, IL-2 receptor signaling, GRAIL, neddylation, phosphor-S6

## Abstract

Autoimmune diseases are a diverse group of chronic disorders characterized by inappropriate immune responses against self-antigens, resulting in persistent inflammation and tissue destruction. Affecting an estimated 7–10% of the global population, these conditions include both systemic and organ-specific entities such as systemic lupus erythematosus (SLE), rheumatoid arthritis (RA), type 1 diabetes (T1D), and multiple sclerosis (MS). Despite their clinical heterogeneity, autoimmune diseases share a common etiologic framework involving the convergence of genetic predisposition, environmental exposures, and immune dysregulation. Genome-wide association studies (GWAS) have identified hundreds of risk loci, most notably within the major histocompatibility complex (MHC), and highlighted the role of non-HLA genes regulating cytokine signaling, antigen presentation, and T cell tolerance. The majority of disease-associated variants lie in non-coding regulatory elements, suggesting that transcriptional dysregulation plays a central role in disease susceptibility. Yet, genetics alone does not determine disease onset—environmental factors such as infections, diet, microbiome alterations, and hormonal influences critically shape immune responses and may trigger disease in genetically susceptible individuals. Additionally, epigenetic modifications further compound these effects, creating lasting changes in gene expression and immune cell function. At the core of autoimmune pathogenesis lies immune dysregulation, particularly failure of peripheral tolerance maintained by regulatory T cells (Tregs). While Treg frequencies may appear normal in patients, emerging data indicate intrinsic signaling defects—especially impaired IL-2 receptor (IL-2R) signal durability—compromise Treg suppressive function. This dysfunction is linked to aberrant degradation of key IL-2R second messengers, including phosphorylated JAK1 and DEPTOR, due to diminished expression of GRAIL, an E3 ligase that inhibits cullin RING ligase activation. This review integrates recent insights across genetic factors, environmental triggers, and immune dysregulation to build a comprehensive understanding of autoimmune disease pathogenesis. We propose a novel therapeutic strategy targeting IL-2R signaling using Neddylation Activating Enzyme inhibitors (NAEis) conjugated to IL-2 or anti-CD25 antibodies. This approach selectively restores Treg function and immune tolerance without inducing systemic immunosuppression. By focusing on immune restoration rather than suppression, This therapy could provide an off the shelf therapy for many different autoimmune diseases.

## Introduction

Autoimmune diseases affect an estimated 15 million individuals in the United States and represent a significant burden of chronic morbidity (1). These disorders arise when the immune system, which normally protects the host from pathogens, mistakenly attacks self-antigens, leading to sustained inflammation and tissue damage. This loss of immune tolerance stems from the failure of central and peripheral regulatory mechanisms that normally eliminate or restrain autoreactive T and B lymphocytes (2). Over 80 autoimmune disorders have been identified, varying in target organs, immunopathology, and clinical progression (3). Among the most prevalent are rheumatoid arthritis (RA), psoriasis, autoimmune thyroid disease, systemic lupus erythematosus (SLE), and type 1 diabetes (T1D) (1). Despite their heterogeneity, these diseases often share core immunologic features, such as autoreactive lymphocytes and/or pathogenic autoantibodies. For instance, diseases such as Graves’ disease, myasthenia gravis, and pemphigus vulgaris are classic examples of autoantibody-mediated disorders (4), whereas multiple sclerosis (MS) and T1D are primarily driven by autoreactive T cells-mediated tissue destruction (5).

Autoimmune diseases are widely recognized as multifactorial, arising from a complex interplay of genetic predisposition, environmental triggers, and immune dysregulation (6, 7). Genetically, these disorders show familial aggregation and comorbidity pointing to shared heritable risk factors. Genome-wide association studies (GWAS) have elucidated the polygenic nature of autoimmunity and revealed extensive overlap in risk loci across diseases (8). Several polymorphisms, particularly within the human leukocyte antigen (HLA) locus, which influence antigen presentation, confer this heightened risk of autoimmune diseases. In addition, non-HLA loci that affect cytokine signaling, lymphocyte activation, and immune regulation are involved (9). Hundreds of susceptibility loci have been found across autoimmune diseases, underscoring shared patho-mechanisms such as impaired antigen presentation and checkpoint dysregulation (10). However, most of these variants are located in non-coding regions, complicating functional interpretation and therapeutic strategies (11). Integrative approaches combining GWAS with transcriptomic, epigenomic, and single-cell approaches are increasingly important for uncovering causal variants and cell-specific regulatory circuits.

Sex-based differences represent one of the most consistent and striking features of autoimmune disease epidemiology, with female accounting for nearly 80% of all cases (12). This disparity is attributed to a combination of hormonal, genetic, and immunological factors. Estrogens enhance humoral response and support autoreactive B cell survival, while androgens generally suppress immune activation (13). Furthermore, several immune-related genes located on the X chromosome—some of which escape X-inactivation—may contribute to heightened immune reactivity in females (12). These sex differences not only influence disease prevalence but also affect therapeutic responses and outcomes in clinical trials. Yalcinkaya et al. have comprehensively outlined the biological and clinical challenges that are unique to each sex (14). These differences are thought to reflect evolutionary pressures that have shaped immune function differently in males and females. For example, the female immune system must remain flexible to tolerate pregnancy while still maintaining effective immune defenses, all within the constraints of metabolic efficiency (15). Consequently, females generally exhibit stronger innate and adaptive immune responses at baseline compared to males, leading to faster infection clearance, enhanced vaccine efficacy, and more robust antibody responses (16). This immunological advantage also extends to survival outcomes; males experience higher mortality following infection, even when age is accounted for, whereas females often mount more vigorous humoral responses, cytokine production, and T cell activation in response to immune challenges (17). Another example is the role of sex hormones in the development and progression of allergic asthma, that varies with age, particularly between childhood, adolescence, and adulthood. During childhood, boys are consistently at higher risk for developing asthma, possibly due to differences in lung and airway growth, where female lungs tend to be smaller and has been observed to weigh less at necropsy than male lungs (18, 19). However, no major differences in asthma symptom severity are observed between boys and girls during this stage (20).

Environmental exposures also play a pivotal role in triggering or modulating autoimmune responses. Infectious agents are well-established contributors to disease risk and progression. Viruses, such as Epstein-Barr virus (EBV), are implicated in systemic lupus erythematosus (SLE), rheumatoid arthritis (RA), and Sjögren’s syndrome (21). While EBV infection is nearly ubiquitous and often asymptomatic, its interaction with host genetic background, alongside dysregulated immune homeostasis, may precipitate disease in susceptible individuals (22). Similarly, post-infectious autoimmune manifestations have been increasingly reported following SARS-CoV-2 infection, including Guillain–Barré syndrome, antiphospholipid syndrome, and systemic autoimmunity (23). Nonetheless, the relatively low incidence of post-infectious autoimmunity and incomplete concordance among monozygotic twins underscore the importance of environmental triggering events and immune regulation (24, 25).

Dietary factors, though often underappreciated, emerge as key modulators of risk of autoimmunity. For example, Crohn’s disease is not classified as a gluten-sensitive enteropathy; gluten has been shown to exacerbate intestinal inflammation in selective individuals (26). In such cases, clinical improvement following gluten exclusion suggests a partially reversible inflammatory process. Other dietary antigen-specific disorders, such as peanut allergy and celiac disease, reflect antigen-specific mucosal immune dysregulation and epithelial barrier integrity. These conditions often feature aberrant T cell responses to dietary antigens, shaped by host genetics and microbiota composition, with downstream consequences for systemic autoimmunity (25). Beyond diet, modifiable lifestyle factors such as obesity significantly influence autoimmune disease development and progression. Adipose tissue functions as an immunologically active organ, releasing proinflammatory cytokines and adipokines such as IL-6 and leptin. These mediators promote Th17 differentiation and impair regulatory T cell (Treg) function, and contribute to autoantibody production, epitope spreading, and chronic inflammation (27). While genetic and environmental risk factors are largely non-modifiable, the immune system, particularly regulatory T cells (Treg), remains an actionable therapeutic target. Tregs are essential for maintaining peripheral tolerance and blocking autoimmunity, yet their dysfunction is increasingly implicated in the pathogenesis of autoimmune diseases.

Current therapeutic strategies for autoimmune diseases face several limitations: (i) limited efficacy, as most treatments mitigate symptoms without correcting the underlying immunologic defect; (ii) a compartmentalized, organ-specific approach to care, which hinders the development of unified therapies; (iii) lack of specificity, with broad immunosuppression increasing the risk of infections and malignancy; and (iv) long-term toxicity from chronic immunosuppressive and corticosteroid use. These challenges underscore the urgent need for more precise and mechanism-based therapies. While many risk factors are non-modifiable, the immune system—and particularly Tregs—remains a viable and actionable therapeutic target. Tregs are central to maintaining peripheral tolerance, and their dysfunction has emerged as a critical driver of autoimmune pathogenesis. Despite advances in immunomodulatory therapies, many interventions have yielded limited efficacy due to immune redundancy and disease heterogeneity.

**Figure 1:**
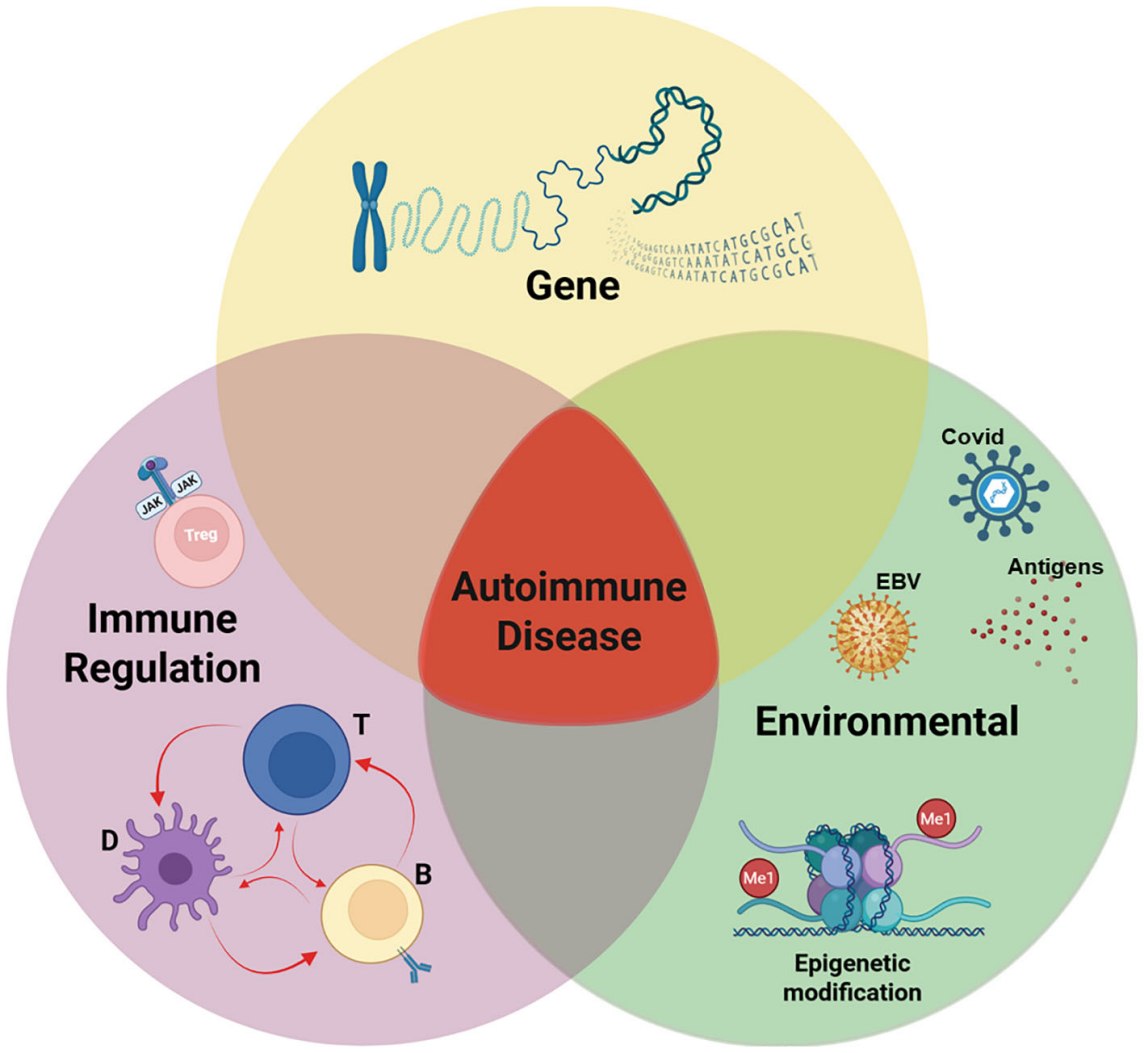
Pathogenesis of autoimmune disease. Our working hypothesis is that the development of an autoimmune disease arises when three critical factors converge: genetic predisposition, exposure to an environmental trigger, and defects in immune regulation. In a genetically predisposed individual, the immune system’s response to an environmental pathogen, combined with defects in regulatory mechanisms, leads to autoimmunity. While the relative influence of each component may differ across individuals and specific diseases, the onset of autoimmune disease typically requires the intersection of all three elements, as illustrated in the Venn diagram. Among this triad, a defect in immune regulation is the only rational target for therapy. Our studies (described below) have identified a correctable defect in interleukin 2 receptor (IL-2R) signaling of regulatory T cells (Tregs) from patients with autoimmune diseases and allergic asthma. Created with BioRender.com.

These limitations underscore the need for precision medicine approaches that integrate immunophenotyping, biomarker-guided patient selection, and rational combination strategies. In this review, we explore the multifactorial origins of autoimmune diseases, with a focus on genetic susceptibility, environmental triggers, and immune dysregulation (**Figure 1**). We emphasize emerging insights into Treg dysfunction and discuss how these mechanisms are informing the development of targeted therapeutic interventions.

## Genetic susceptibility in autoimmune disease

Genetic predisposition is a key determinant in the development of autoimmune diseases and provides foundational insight into the mechanisms underlying immune dysregulation. Familial clustering of autoimmune conditions and strong associations with specific gene loci underscore the role of inherited susceptibility. Genome-wide association studies (GWAS) have revolutionized our understanding of the genetic architecture of autoimmunity, identifying hundreds of susceptibility loci shared across multiple autoimmune conditions and confirming their polygenic nature and overlapping immunopathology.

Among these loci, the major histocompatibility complex (MHC)—particularly HLA class II alleles—demonstrate the strongest and most consistent associations. For example, HLA-DRB1 variants are linked to rheumatoid arthritis (RA), and HLA-DR3 and HLA-DR4 significantly increase the risk for type 1 diabetes (T1D) (28). These alleles influence antigen presentation to CD4^+^ T cells and shape the T cell repertoire during thymic selection. Sequence analyses have revealed that susceptibility is not conferred by major structural mutations, but rather by subtle variations in the peptide-binding grooves of MHC molecules that affect the presentation of self-antigens and promote autoreactivity (29–31). Beyond HLA, numerous non-MHC genes contribute to autoimmune risk. Notable examples include: PTPN22, encoding a tyrosine phosphatase that negatively regulates TCR signaling; STAT4, a transcription factor involved in IL-12-mediated Th1 differentiation; and CTLA4, an immune checkpoint molecule that dampens T cell activation (28). Although these variants confer measurable risk, their penetrance is often low, reinforcing the complexity of autoimmune pathogenesis and the necessity of considering gene–environment interactions. A striking feature of autoimmunity genetics is that over 90% of disease-associated variants map to non-coding regions of the genome, primarily enhancers and regulatory elements, rather than protein-coding genes (32). These findings suggest that most of the autoimmune risk is mediated through transcriptional regulation rather than protein dysfunction. However, functional interpretation of non-coding variants is challenging due to linkage disequilibrium (LD) and the presence of multiple correlated variants within associated loci (33).

Recent advances have begun to address this challenge through fine-mapping, chromatin profiling, and multi-omics integration. For example, a study analyzing 21 autoimmune diseases found that approximately 60% of putative causal variants colocalize with super-enhancers marked by H3 lysine 27 acetylation (H3K27ac) in CD4+ T cell populations and B lymphoblastoid cells, including stimulus-responsive enhancers that produce enhancer RNAs (eRNAs) (32). Many variants act by disrupting transcription factor binding, such as NF-κB, thereby altering gene expression programs that regulate immune cell differentiation and function. A case in point is the Crohn’s disease–associated variant rs61839660, which links to stimulation-dependent intronic interleukin-2 (IL-2) receptor alpha (IL2Rα) enhancer. CRISPR activation screens demonstrated that this risk allele impairs the timing of IL2Rα (CD25) induction upon T cell stimulation (20). Given that IL-2 signaling is critical for FOXP3^+^ regulatory T cell (Treg) maintenance, such regulatory perturbations may undermine Treg-mediated tolerance and facilitate autoimmunity. The IL2Rα locus is implicated in several autoimmune diseases, with risk alleles sometimes exerting opposing effects across conditions (34). In addition to susceptibility alleles, certain protective variants can modulate disease severity or delay onset. This phenomenon has been documented in murine lupus models, where protective loci counteract the expression of high-risk alleles (35). Identifying such variants may reveal new therapeutic targets capable of restoring immune tolerance.

The MHC region remains the most robust genetic contributor to autoimmunity, influencing both central tolerance (T cell selection in the thymus) and peripheral activation of autoreactive T cells. Yet, the observation of RA and T1D in individuals lacking classic MHC-risk haplotypes highlights the presence of alternative genetic and regulatory mechanisms (36, 37). Moreover, conserved sequence motifs—such as the shared epitope in HLA-DRB1—further support the idea that specific peptide presentation characteristics are central to disease susceptibility (30). Despite these advances, the interpretation of genetic data remains complicated by the polygenic and pleiotropic nature of autoimmunity. Some risk alleles are shared among diseases yet exert divergent functional outcomes, exemplified by variable responses to TNF-α inhibitors across different autoimmune conditions. Such complexities necessitate refined approaches that incorporate context-specific regulatory information, ideally at single-cell resolution in human disease tissues.

The promise of genetics in clinical translation is increasingly evident. Functional studies are beginning to elucidate the molecular impact of key variants, and the development of high-throughput CRISPR-based assays will accelerate this progress. Importantly, genetic findings are leveraged to inform drug development, as evidenced by the fact that nearly two-thirds of drugs approved by the FDA in 2021 were supported by genetic evidence (38). In parallel, polygenic risk scores (PRS) are being developed to identify individuals at elevated risk for autoimmune diseases, enabling presymptomatic screening and preventive intervention trials, such as those targeting T1D (39, 40).

## Environmental triggers of autoimmune disease

While genetic predisposition plays a fundamental role in autoimmune disease susceptibility, it is insufficient to fully explain disease onset. This is evidenced by the relatively low concordance rates of autoimmune diseases among monozygotic twins, typically less than 50%, highlighting the essential contribution of environmental and epigenetic factors to disease initiation and progression. Environmental exposures, in combination with stochastic immune events and a susceptible genetic background, shape individual risk profiles and influence the trajectory of autoimmunity. Factors such as infections, diet, gut microbiota, hormonal influences, and random immune receptor recombination all contribute to disease heterogeneity and onset.

Stochastic immune variation, such as random T cell receptor (TCR) rearrangements during thymic development and differential antigen exposure in the periphery, results in unique immune repertoires even among genetically identical individuals. These differences may enable autoreactive clones to evade central tolerance and respond aberrantly to environmental antigens (34). Among the environmental triggers, infectious agents are among the most extensively studied. Pathogens can initiate or exacerbate autoimmune responses through mechanisms including molecular mimicry, bystander activation, and epitope spreading (35). One well-characterized example is Lyme arthritis, caused by Borrelia burgdorferi, a tick-borne spirochete. In approximately 10% of affected individuals, arthritis persists long after the infection has resolved, and evidence suggests an autoimmune response against leukocyte function-associated antigen-1 (LFA-1), a self-protein that shares sequence homology with the bacterial outer surface protein (36). Similarly, in MS, epidemiological data point to viral infections as potential triggers, though no single causative agent has been conclusively identified (37). In systemic lupus erythematosus (SLE), a particularly compelling association has been made with Epstein-Barr virus (EBV). Longitudinal studies in military cohorts have shown that individuals who later developed SLE exhibited elevated titers of EBV-specific antibodies years before clinical onset. EBV nuclear antigens such as EBNA1 exhibit molecular similarity to host antigens, including the Smith (Sm) protein, potentially initiating an autoimmune cascade through molecular mimicry (16).

In addition to infections, the gut microbiome plays a crucial role in modulating immune responses and maintaining mucosal tolerance. Alterations in microbial composition (dysbiosis) can compromise regulatory T cell (Treg) function, disrupt intestinal barrier integrity, and promote systemic inflammation. Emerging evidence suggests that dietary antigens, particularly gluten and other food allergens, may contribute to disease onset by enhancing intestinal permeability and promoting aberrant immune activation in genetically susceptible individuals (25).

Nutritional exposures, especially during early development, also influence autoimmune risk. Notably, elevated dietary iron intake has been linked to the development of type 1 diabetes (T1D) in children. Observational studies have associated high iron levels in neonatal blood, use of iron-fortified formulas in infancy, prenatal iron supplementation, and high red meat consumption with an increased risk of T1D development (41–44). In our own studies using non-obese diabetic (NOD) mice, we demonstrated that excess dietary iron significantly increases the incidence of hyperglycemia. This effect was attributed to inflammation-induced disruption of iron homeostasis in pancreatic islets. Supporting this connection, Voss et al. recently demonstrated that disease severity in systemic lupus erythematosus (SLE) correlated with transferrin receptor (CD71) expression on TH17 cells. In SLE patients with activated T cells, CD71 promotes excessive iron uptake through enhanced endosomal recycling (45). Because the human body lacks an efficient mechanism for eliminating excess iron, chronic iron overload leads to accumulation in pancreatic β cells, where it catalyzes the generation of reactive oxygen species (ROS). The resulting oxidative stress contributes to β cell apoptosis and autoimmune activation (46). This model underscores how environmental triggers, such as diet, can synergize with genetic predisposition and immune dysregulation to promote autoimmune disease. In this context, T1D represents a prototypical example of how inflammation, immune-genetic factors, and dietary exposure converge to breach immune tolerance and initiate autoimmunity.

Environmental factors may also induce long-lasting changes in gene expression through epigenetic mechanisms, such as DNA methylation and histone modification. These modifications can activate latent susceptibility genes or repress protective regulatory loci, thereby altering immune cell function and promoting autoimmune reactivity. Additionally, hormonal influences—especially differences in sex hormones such as estrogen and testosterone—are known to modulate immune responses and contribute to the marked female predominance observed in many autoimmune diseases.

Despite the growing body of evidence linking environmental exposures to autoimmunity, identifying causative agents remains challenging. Disease onset is often preceded by a prolonged preclinical phase, during which subclinical immune alterations, including the presence of autoantibodies and cytokine imbalances, may be detectable years before symptoms occur. Longitudinal cohort studies using serial sampling have revealed these early signatures, emphasizing the need for enhanced epidemiologic surveillance tools and immune monitoring platforms (47). Integrating high-resolution immune profiling with detailed environmental exposure data may help unravel early pathogenic mechanisms and inform targeted preventive strategies.

## Immune dysregulation in autoimmune disease

Autoreactivity is not, by itself, pathologic. As Lipsky et al. (2001) noted, autoreactivity is an intrinsic feature of T cell biology. All peripheral T cells are positively selected on self–peptide–MHC complexes in the thymus and require continuous interaction with self-MHC in the periphery for survival and function (48). Thus, every mature T cell carries some degree of autoreactive potential. Indeed, autoreactive cells and low-titer autoantibodies are commonly detected in healthy individuals and more frequently in siblings of autoimmune patients (7). Therefore, the presence of autoreactive lymphocytes is necessary but not sufficient to cause disease. Autoimmunity arises when peripheral tolerance mechanisms, which normally restrain pathogenic self-reactivity, fail.

Several tolerance mechanisms exist in the periphery, including clonal deletion, anergy, immune deviation, ignorance, and active suppression by regulatory T cells (Tregs) (49). However, the relative contribution of each mechanism to human autoimmune disease remains incompletely understood. Importantly, experimental models demonstrate that pathogenic autoreactive lymphocytes are present in normal animals and can induce disease under specific conditions, such as in experimental autoimmune encephalomyelitis (EAE) or collagen-induced arthritis (50).

Central tolerance is mediated in part by the transcription factor AIRE (autoimmune regulator), which promotes expression of tissue-specific antigens (TSAs) in medullary thymic epithelial cells, allowing negative selection of high-avidity self-reactive T cells. This is exemplified by the monogenic disorder autoimmune polyendocrine syndrome type 1 (APS-1), caused by mutations in the *AIRE* gene. Patients with APS-1 develop multiple autoimmune disorders, including type 1 diabetes (T1D), adrenal insufficiency, hypothyroidism, vitiligo, and alopecia, due to impaired central deletion of autoreactive T cells (51). Complementing these insights, studies of monogenic disorders affecting peripheral tolerance have also been highly instructive. Autoimmune lymphoproliferative syndrome (ALPS), caused by mutations in the FAS gene, highlights the importance of activation-induced cell death (AICD) in regulating lymphocyte homeostasis (52). In ALPS, failure to eliminate activated T cells leads to lymphoproliferation and systemic autoimmunity (53). Similarly, immune dysregulation, poly-endocrinopathy, enteropathy, X-linked (IPEX) syndrome—a rare, often fatal neonatal autoimmune disorder—is caused by mutations in the transcription factor FOXP3, which is critical for Treg development and function. IPEX illustrates the indispensable role of regulatory T cells in enforcing peripheral tolerance (54).

Other monogenic immune disorders have further expanded our understanding of Treg-dependent tolerance. For example, LRBA (LPS-responsive and beige-like anchor protein) deficiency, CD25 (IL-2Rα) deficiency, and gain-of-function mutations in STAT3 all impair Treg cell development, stability, or suppressive function, and result in syndromes marked by multi-organ autoimmunity (55, 56). These rare syndromes serve as natural models to dissect the molecular checkpoints required to maintain immune tolerance.

Mouse models have recapitulated many of these mechanisms. For instance, Fas-deficient (lpr) and FasL-deficient (gld) mice develop a lupus-like autoimmune lymphoproliferative disorder analogous to ALPS. CTLA-4 knockout mice (CTLA-4^−^/^−^) exhibit rapid-onset, fatal lymphoproliferation with massive immune cell infiltration, reflecting the critical role of CTLA-4 in attenuating T cell responses. CTLA-4 polymorphisms have been associated with human autoimmune thyroid disease and T1D (57). Similarly, IL-2^−^/^−^ and CD25^−^/^−^ mice develop fulminant autoimmune disease characterized by lymphoproliferation, colitis, and autoantibody production (58), reinforcing IL-2’s non-redundant role in immune regulation.

While these early phenotypes were attributed to a lack of apoptosis or costimulatory control, they are now understood to reflect regulatory T cell deficiency. CD4^+^CD25^+^FoxP3^+^ Tregs are essential for maintaining peripheral tolerance, and their depletion leads to spontaneous autoimmune disease in both mice and humans (59). Conversely, adoptive transfer of Tregs prevents disease in susceptible models (60). Tregs employ multiple mechanisms of suppression, including secretion of IL-10, TGF-β, and IL-35, consumption of IL-2 to starve effector cells, CTLA–4–mediated modulation of antigen-presenting cells, and suppression of T cell proliferation. Notably, Treg stability and function are critically dependent on IL-2 signaling. CD25 is not merely a surface marker but mediates the high-affinity IL-2R signal that is essential for Treg homeostasis. This explains why CD25 or IL-2 deficiency disrupts Treg populations and leads to systemic autoimmunity.

More recently, the Notch signaling pathway has emerged as a key modulator of Treg biology and a potential contributor to immune dysregulation. Notch family receptors, particularly Notch1 and Notch4, exert divergent effects on Treg function depending on cellular context and immune environment. Notch1 signaling supports the survival and suppressive capacity of CD4^+^ Tregs, partly by regulating autophagy under cytokine-deprived conditions. Inactivation of Notch1 leads to impaired Treg-mediated suppression and increased effector T cell activation, exacerbating autoimmune pathology. In contrast, Notch4 signaling has been shown to impair CD8^+^ Treg function, particularly in inflammatory conditions. Elevated Notch4 activity correlates with diminished suppression and increased vascular inflammation. Inhibition of Notch4 restores Treg function and reduces tissue inflammation. These findings underscore the context-dependent and receptor-specific roles of Notch signaling in immune regulation. Enhancing Notch1 activity may bolster Treg stability and therapeutic efficacy in autoimmune diseases, whereas targeted inhibition of Notch4 could mitigate inflammation and restore tolerance. Thus, the Notch pathway represents a promising axis for therapeutic intervention in immune-mediated diseases.

Together, these studies illustrate how immune dysregulation—whether through failed central tolerance, defective Treg function, or disrupted apoptotic or signaling pathways—lies at the core of autoimmune pathogenesis. Insights from both experimental models and rare monogenic disorders have provided a blueprint for targeting the regulatory immune networks essential for maintaining tolerance.

### Regulatory T cell dysfunction and IL-2 signaling

**Figure 2:**
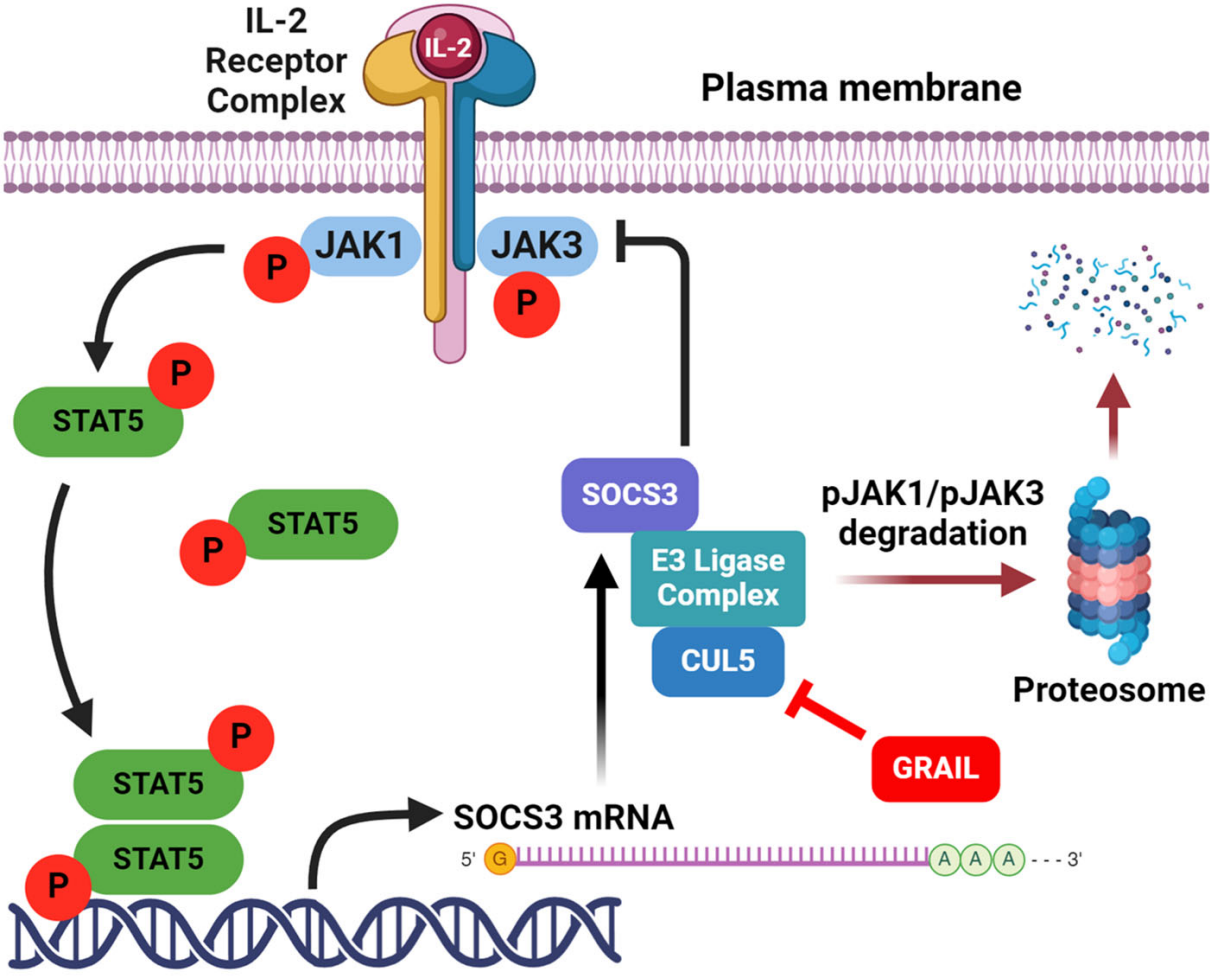
Activation of Tregs by low-dose IL-2. Low-dose IL-2 activates the high affinity IL-2R to phosphorylate JAK1, that then phosphorylates STAT5 to drive the expression of genes required for Treg function. Created with BioRender.com.

While Treg numbers may be modestly decreased in some autoimmune conditions, emerging evidence suggests a dominant role for functional defects. Clinical trials using low-dose IL-2 to selectively expand Tregs have shown increased Treg frequency, yet limited clinical efficacy, implying preserved quantity but impaired function (61). Recent studies identify a defect in IL-2 receptor (IL-2R) signaling desensitization in Tregs from autoimmune patients (62). Normally, IL-2R signaling is tightly regulated through a feedback loop involving the degradation of activated JAK1 kinase by cullin5 (CUL5)-based E3 ligase complexes. Neddylation of CUL5 at lysine 724 (K724) untethers the E2 transferase bound by an RBX protein at the carboxy terminus of CUL5. This allows ubiquitination and degradation of phosphorylated JAK1, terminating STAT5 activation (**Figure 2**). However, in healthy Tregs, this process of neddylation is competitively inhibited by GRAIL (Gene Related to Anergy in Lymphocytes), an E3 ligase that mono-ubiquitinates CUL5, also at K724 (62, 63). This ubiquitination competitively inhibits the requisite neddylation of lysine 724 and thus CRL-mediated degradation of pJAK1, prolonging IL-2R signaling and thus maintenance of pSTAT5 expression. Prolonged pSTAT5 expression promotes transcription of Treg signature genes, including Foxp3. GRAIL may also ubiquitinate lysine 720 on CUL1 and block the CRL degradation of DEPTOR, a negative regulator of mTOR, to maintain Treg stability (64).

**Figure 3:**
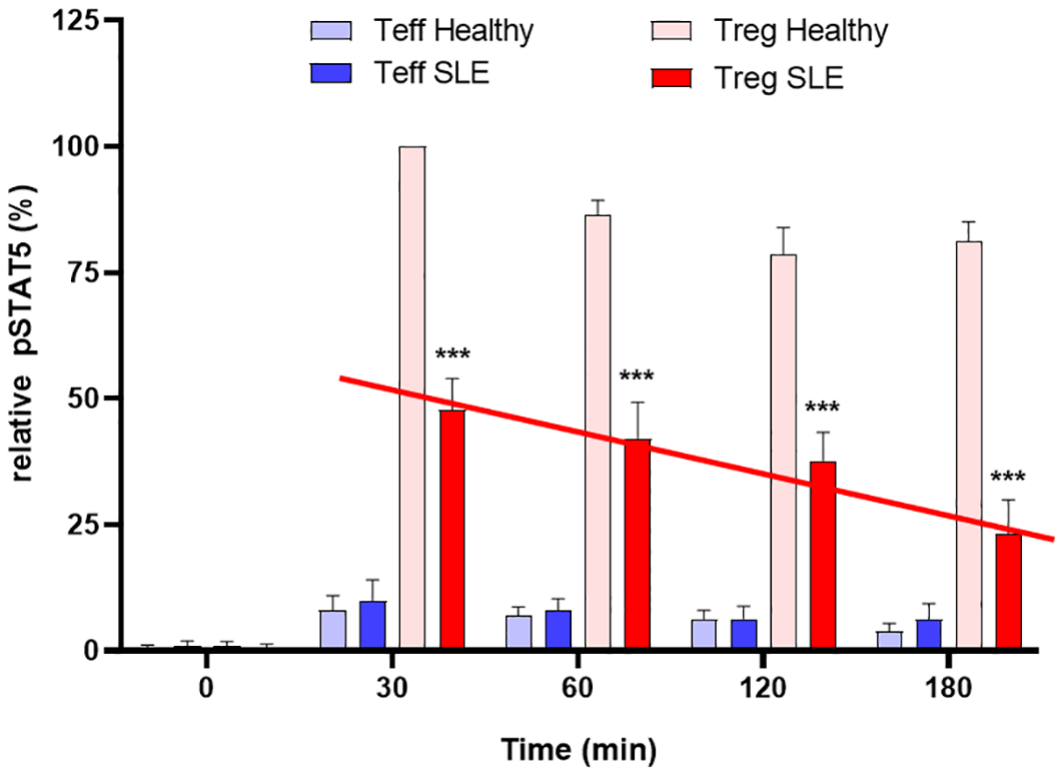
Defective inhibition of IL-2 desensitization in the Tregs of SLE patients. The 25 SLE patients studied were selected from a clinic in Mexico City, irrespective of current disease status. These graphs represent Western blot data of pSTAT5 phosphorylation in Teffs and Tregs of the SLE patients and healthy sex and age-matched controls after stimulation with low dose IL-2 (1 ng/ml; 25 IU/ml) for the indicated amounts of time. Expression is shown as a percentage of pSTAT5 measured from 30 min-stimulated control Tregs. A defect in the inhibition of IL-2R desensitization is observed in the Tregs of SLE patients (red line) n=6 per group ***p < 0.001 by 2-way ANOVA. Fathman et al. (62).

In contrast, Tregs from patients with autoimmune disease exhibit premature desensitization of IL-2R signaling. This leads to rapid degradation of pJAK1 and DEPTOR, resulting in impaired pSTAT5 activity, mTOR dysregulation, and reduced Treg suppressive function. Notably, Tregs from SLE patients maintain IL-2 stimulated pSTAT5 expression for only ~1.5 hours compared to >3 hours in healthy controls, indicating a failure to sustain the transcriptional program required for immune regulation (**Figure 3**). This mechanistic defect highlights the importance of not expanding Treg numbers but of restoring IL-2R signal fidelity. Therapeutic strategies aimed at stabilizing IL-2R signaling, via GRAIL modulation, inhibition of premature CUL5 neddylation, or DEPTOR preservation, may represent promising interventions to restore immune tolerance in autoimmunity.

### Neddylation of cullin RING ligases is a promising molecular target to restore defective IL-2R signaling in Tregs

We recently identified a druggable defect in the IL-2R signaling pathway of Tregs from patients with autoimmune diseases (62). This defect in IL-2R second messenger stability impairs the function of Tregs, which are crucial for maintaining immune tolerance to prevent autoimmunity. Specifically, patients with autoimmune diseases have diminished expression of GRAIL (Gene Related to Anergy in Lymphocytes), a novel ubiquitin E3 ligase that acts as a competitive inhibitor of neddylation (activation) of the cullin5 (CUL5) ring ligase (CRL) (63). GRAIL inhibits CUL5-mediated degradation of phosphorylated JAK1 (pJAK1), a second messenger downstream of IL-2R activation. GRAIL normally mono-ubiquitinates CUL5 lysine K724 (65), which prevents K724 neddylation and results in CUL5 inhibition, but in autoimmune patients with diminished GRAIL expression, increased CUL5 activity leads to a more rapid pJAK1 degradation. Reduced pJAK1 expression diminishes phosphorylated STAT5 (pSTAT5), which is essential for the transcription of the genes required for Treg function.

**Figure 4:**
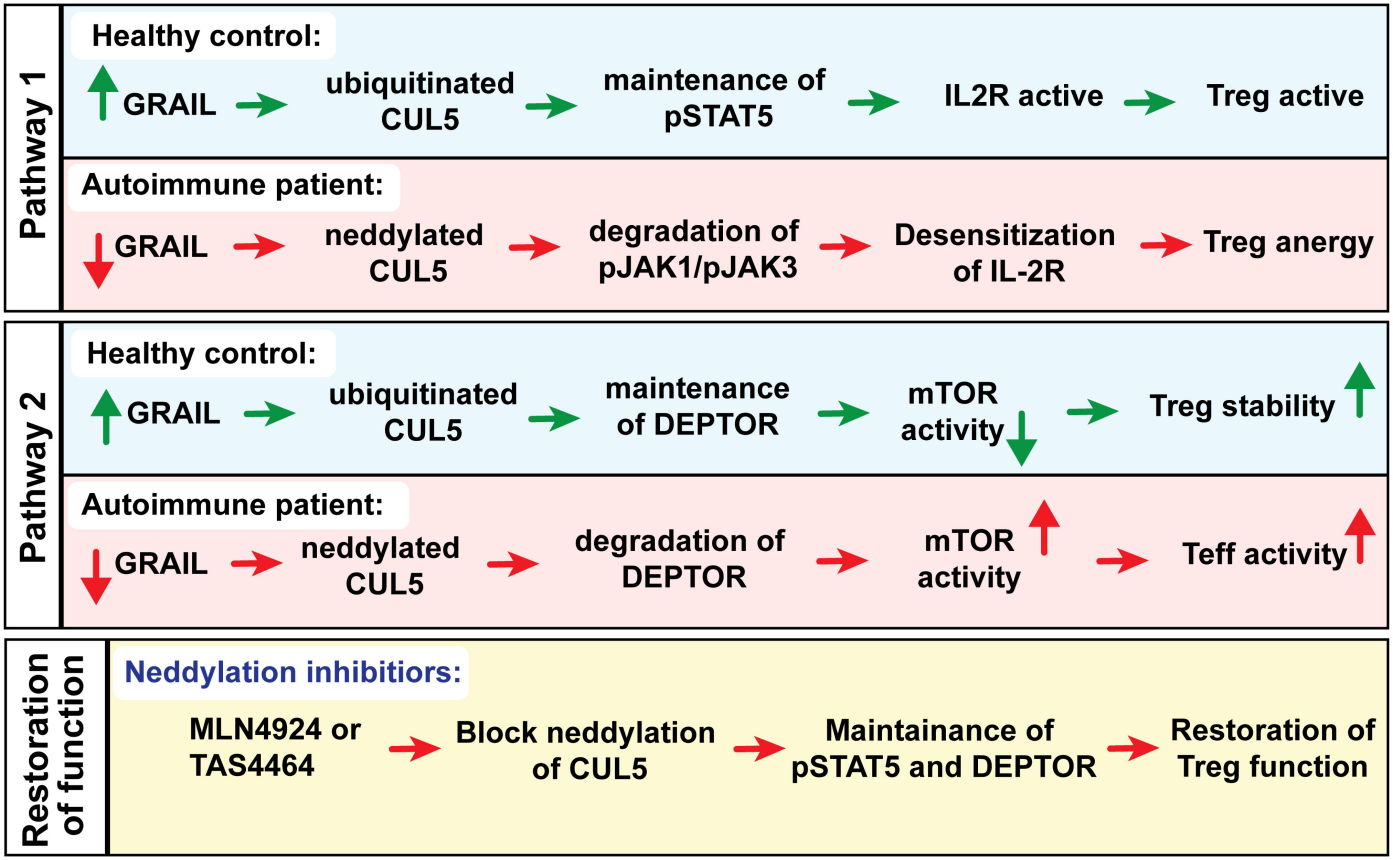
Could a neddylation activating enzyme inhibitors (NAEi) such as MLN4924 or TAS4464 replace GRAIL to treat autoimmunity?

In addition to elevated degradation of pJAK1 by CUL5, diminished GRAIL also leads to reduced DEPTOR (DEP Domain Containing mTOR Interacting Protein) expression. DEPTOR is an endogenous inhibitor of mTOR activation that functions in a manner similar to the mTOR inhibitor and immunosuppressant rapamycin, although through a distinct mechanism of action. Sustained pSTAT5 activity in healthy donor Tregs allows the genes for Treg function to be transcribed, leading to activation of regulatory function, whereas the maintenance of DEPTOR supports the Treg phenotype. In autoimmune patients, similar activation of Tregs with low dose IL-2 results in only transient activation of IL-2R signaling due to a defect in the inhibition of IL-2R desensitization, resulting in reduced pSTAT5 and DEPTOR degradation and loss of the Treg phenotype (**Figure 4**). Therefore, we investigated CUL5 neddylation as a molecular target that leads to the desensitization (turning off) of IL-2R signaling in Tregs. We showed that restoring the inhibition of the CUL5 second messenger degradation pathways with neddylation activating enzyme inhibitor (NAEi) could enhance/restore Treg function and re-establish normal immune tolerance. Reduced GRAIL expression in autoimmune patients leads to diminished Treg function via two distinct pathways that favor the neddylation of CUL5. Neddylation inhibitors restore Treg function.

**Figure 5:**
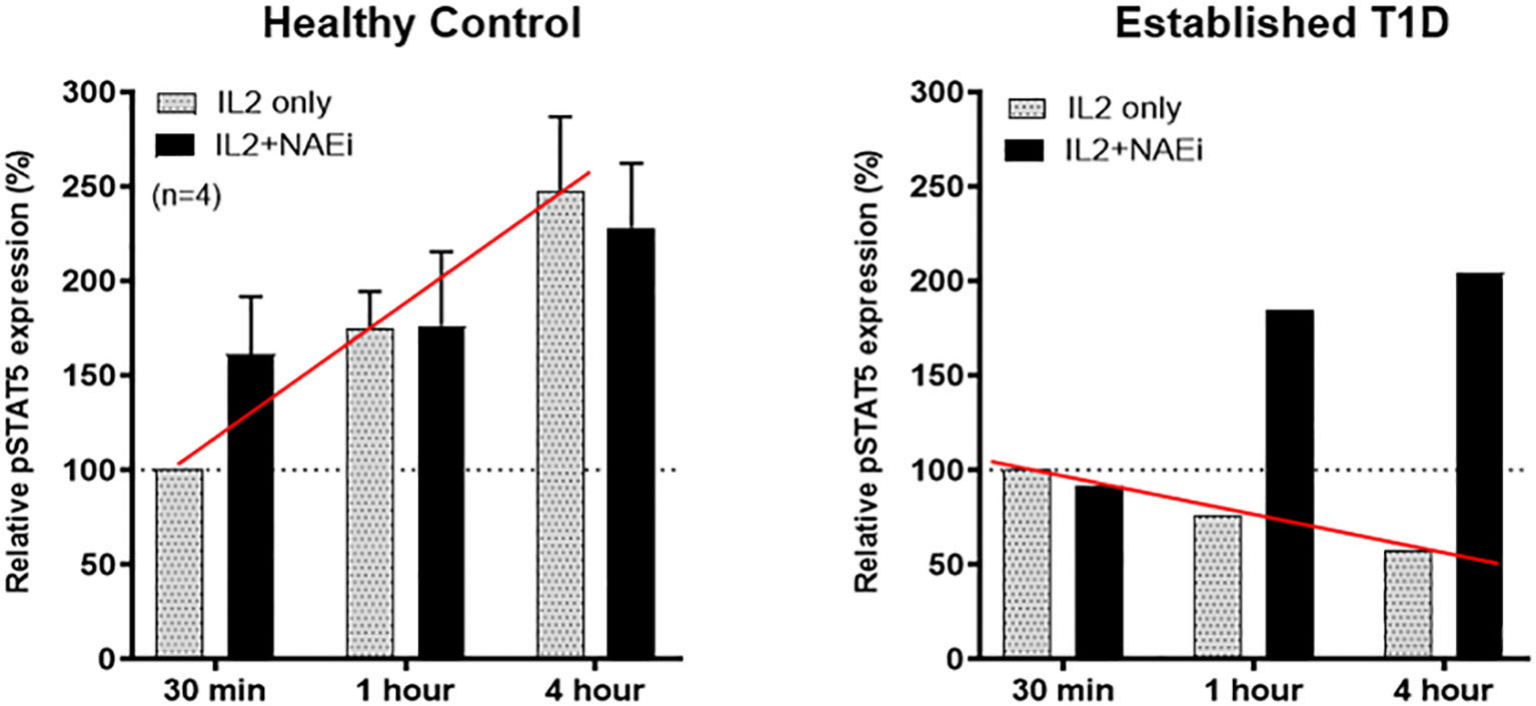
Tregs of healthy controls or an established T1D patient (who also had diminished GRAIL expression) were treated with low dose IL-2 (1 ng/ml) in the presence or absence of an NAEi (MLN4924; 400 uM). Like SLE, low dose IL-2 stimulated pSTAT5 expression is reduced in Tregs of T1D patients vs. controls (red line). Combination treatment of low dose IL-2 and the NAEi increased and prolonged pSTAT5 expression in the Tregs of T1D patients back to control Treg levels (solid black columns). The NAEi corrects for the GRAIL deficiency.

We identified CUL5 as a primary molecular target of NAEi therapy and demonstrated that blocking CRL neddylation using NAEi can restore proper Treg function in human Treg assays *in vitro* and in animal models of autoimmunity *in vivo* (62). In addition to maintaining pSTAT5 signaling, NAEi treatment blocks DEPTOR degradation by CUL1, leading to reduced mTOR activity. mTOR is a serine/threonine kinase that is present in almost all eukaryotic cells. It can form two distinct complexes, mTORC1 and mTORC2, with separate functions. While mTOR activity is essential for Treg function, excessive activation of mTORC1 can impair their suppressive capacity and compromise their stability. Previous studies have already shown that DEPTOR stabilizes FOXP3 expression and enhances Treg function (**Figure 5**). mTORC1 promotes growth and metabolism, particularly glycolysis, in Teff. In contrast, Tregs are more metabolically efficient and less dependent on mTORC1, which partly explains their survival under mTORC1 inhibition (66). mTORC2, on the other hand, regulates Akt signaling, which is important for Treg survival and migration. However, its role in Tregs is more complex. While mTORC2 is supportive of Treg function, overactivation can reduce their suppressive abilities. Thus, mTORC1 can be seen as unnecessary for Tregs, while mTORC2 functions as a critical rheostat of their activity (67). Tregs naturally have lower mTOR activity compared to Teff cells. They express FOXP3, which promotes the expression of Pim-2, a growth kinase that operates independently of mTOR. This allows Tregs to maintain a functional immune response while minimizing their reliance on mTOR-dependent growth pathways. Additionally, Tregs upregulate PTEN, which inhibits the PI3K pathway and further suppresses mTOR activity. As a result, Tregs are able to activate STAT5 signaling in response to IL-2 without mTOR dependence. As mTOR is a major determinant of Treg function, mTOR signaling must be tightly regulated. Regulation is done through a feedback loop where mTOR levels control GRAIL stability through Otubain-1 (Otub1) expression (68). In Teff cells, IL-2 binding to its receptor activates the PI3K/Akt pathway, leading to mTORC1 activation. This pathway also upregulates Otub1, a deubiquitinase that allows GRAIL degradation by deubiquitinating USP8, promoting Teff cell proliferation. Otub1 destabilizes GRAIL by deubiquitinating USP8, the deubiquitinase that deubiquitinates autoubiquitinated GRAIL (68). In Tregs, maintaining low mTOR activity through DEPTOR and GRAIL-mediated inhibition is critical. IL-2R signaling is maintained through mTOR suppression via Otubain-1 alternative reading frame 1 (Otub1-ARF1) stabilization of GRAIL. Otub1-ARF1 is a catalytically inactive form of Otub1 and competitively inhibits Otub1 by competing for binding to GRAIL and allowing ubiquitinated USP8 to bind to and deubiquitinate GRAIL, thus preserving GRAIL levels (69). GRAIL keeps IL-2 receptor (IL-2R) signaling active by blocking neddylation of CUL5. In the absence of GRAIL, CUL5 is neddylated, leading to the degradation of IL-2R signaling proteins such as pJAK1.

GRAIL also plays a crucial role in maintaining Treg function by inhibiting CRL1-mediated ubiquitination of DEPTOR, which keeps mTOR in an inactive state. This inhibition of mTOR activity is important for promoting the stability and suppressive capacity of Tregs. By maintaining low mTOR activation, GRAIL helps Tregs avoid the shift toward a more proliferative and glycolysis-dependent phenotype. However, when GRAIL expression is diminished, DEPTOR degradation allows the activation of mTOR, skewing Tregs toward an effector-like phenotype (62). The activated mTOR pathway further contributes to this shift by upregulating Otub1 (70). This feedback loop exacerbates the loss of GRAIL and amplifies mTOR activation, thereby impairing Treg function and promoting immune dysregulation.

### Can phospho-S6 be a surrogate marker of dysfunctional regulatory T Cells?

The mechanistic target of rapamycin complex 1 (mTORC1) plays a pivotal role in regulating the metabolic and functional state of regulatory T cells (Tregs). A key downstream effector of mTORC1 is ribosomal protein S6, which is phosphorylated by the p70 S6 kinase (S6K1) following mTORC1 activation. The phosphorylated form, phospho-S6 (p-S6), serves as a widely used surrogate marker of mTORC1 activity and has been extensively utilized to assess the signaling and metabolic status of Tregs in both physiological and pathological contexts. In Tregs, basal mTORC1 signaling is essential for survival and homeostatic proliferation. However, excessive or sustained activation of mTORC1, reflected by elevated p-S6 levels, has been linked to impaired suppressive function and reduced lineage stability (71). For instance, an increased p-S6 expression in Tregs has been observed under inflammatory conditions, in tumor microenvironments, and during autoimmune disease progression, often coinciding with diminished FOXP3 expression and acquisition of effector-like properties (72). Conversely, Tregs with low to moderate mTORC1 activity, as indicated by reduced p-S6 levels, typically maintain robust suppressive capacity and transcriptional stability (73). p-S6 is readily detectable by phospho flow cytometry and immunoblotting. It is a valuable tool for real-time monitoring of mTORC1 activation in specific Treg subsets. Moreover, p-S6 levels are modulated by cytokine signaling, particularly interleukin-2 (IL-2), as well as nutrient availability and T cell receptor (TCR) engagement, highlighting the integration of environmental cues in shaping Treg metabolism and function. Pharmacological inhibition of mTORC1 with agents such as rapamycin suppresses p-S6 levels and has been shown to promote the expansion and stability of functional Tregs, underscoring the therapeutic relevance of p-S6 as both a biomarker and a pharmacodynamic readout in immunomodulatory interventions. Therefore, p-S6 could serve as a sensitive and dynamic marker of mTORC1 activation in Tregs, providing key insights into their metabolic programming, functional competence, and therapeutic potential in the context of immune regulation.

## Other immune components in autoimmune diseases beyond regulatory T cells

Although regulatory T cells (Tregs) are essential for maintaining immune tolerance, their dysfunction alone does not fully explain the complexity of autoimmune disease pathogenesis. Increasing evidence demonstrates that multiple immune components interact with or are influenced by defective Treg activity, contributing to disease onset and progression (74). These cellular and molecular elements, particularly B cells, Th17 cells, innate immune cells, and cytokine networks, not only shape the inflammatory environment but also directly modulate or are modulated by Tregs (75). B cells, for example, are not only drivers of autoantibody production but also play regulatory and antigen-presenting roles that intersect with Treg function. Recent studies have highlighted bidirectional communication between B cells and Tregs. Regulatory B cells (Bregs), through the secretion of IL-10, IL-35, and TGF-β, enhance Treg stability and promote their suppressive activity (76). Additionally, naïve B cells have been shown to facilitate the differentiation of inducible Tregs. Specialized subsets such as T follicular regulatory (Tfr) cells and Bregs are crucial for preserving Treg homeostasis in secondary lymphoid organs (77). Conversely, in the context of Treg dysfunction, the balance shifts toward pro-inflammatory B cell activity, leading to heightened antigen presentation, cytokine production (e.g., IL-6, GM-CSF), and the activation of autoreactive T cells. This imbalance is evident in SLE, RA, and T1D, where altered B cell phenotypes are a hallmark of disease. The success of B cell-depleting therapies, such as rituximab and belimumab, supports their key role in autoimmune pathology, particularly when Treg-mediated control is impaired (78).

Moreover, Th17 cells also interact closely with the Treg axis and are frequently expanded in settings of Treg dysfunction. These cells secrete IL-17A, IL-17F, and IL-22, cytokines that drive epithelial disruption, neutrophil recruitment, and chronic inflammation (79). Th17 cells critical to the pathology of MS, RA, psoriasis, and IBD. Furthermore, innate and innate-like immune cells play roles in amplifying inflammation when Treg function is compromised. Dendritic cells (DCs), macrophages, and neutrophils provide antigenic and inflammatory stimuli that prime autoreactive lymphocytes (80). Tregs normally suppress excessive activation of these antigen-presenting cells and limit their cytokine output. In the absence of sufficient Treg control, dysregulated DCs and monocytes can sustain chronic antigen presentation and secrete IL-6, IL-1β, and TNF-α, driving continuous effector T cell activation. These cells are increasingly implicated in the early phases of tissue-specific autoimmune responses and may act upstream of adaptive immunity, particularly in the absence of regulatory restraint. Finally, the cytokine milieu is both a driver and a consequence of Treg dysfunction. Pro-inflammatory cytokines such as IL-6 and IL-23 not only promote Th17 expansion but also destabilize FoxP3 expression in Tregs, compromising their function (81). In contrast, anti-inflammatory cytokines such as IL-10 and TGF-β are required for maintaining Treg identity and suppressive capacity. The imbalance between these opposing cytokine signals creates a feed-forward loop of immune activation and loss of tolerance. Targeted cytokine therapies with IL-6R blockers (e.g., tocilizumab), TNF inhibitors, or IL-23 antagonists have proven effective in restoring immune homeostasis, particularly in cases where Treg function is insufficient to regulate inflammation alone. Together, these findings underscore that while Treg dysfunction is a central feature of many autoimmune diseases, the broader immune context, including aberrant B cell activity, Th17-driven inflammation, dysregulated innate immunity, and cytokine imbalance, converges to shape severe disease pathogenesis. Understanding how these elements interact with Tregs will be essential for developing more comprehensive and durable future immunotherapies.

## Conclusion

Autoimmune diseases are complex, multifactorial disorders involving the interplay of genetics, environmental factors, and immune dysfunction. A comprehensive understanding of autoimmune diseases requires integrating these three critical elements. These components do not act in isolation but interact in complex ways that shape disease susceptibility and expression. Genetically, one of the most well-established risk factors for autoimmune disease lies in specific major histocompatibility complex (MHC) gene variants, which determine how the immune system generates the T cell receptor repertoire to recognize and respond to antigens. These MHC alleles influence the repertoire of peptides presented to T cells, ultimately shaping the adaptive immune response and the threshold for distinguishing self from non-self. In addition to MHC genes, polymorphisms in other immune-regulatory genes, such as those involved in cytokine signaling, costimulatory pathways, and apoptosis, also contribute to an individual’s predisposition to autoimmunity.

The genetic landscape determines not only how the immune system responds to external antigenic challenges but also how effectively it maintains tolerance to self-antigens. Under typical conditions, a balance between immune activation and regulation preserves homeostasis. However, environmental stressors—such as infections, toxins, or changes in the microbiome—can disrupt this balance. These environmental factors may either overwhelm regulatory pathways or trigger aberrant immune responses, particularly when cross-reactivity between microbial and self-antigens occurs. This breakdown in tolerance can initiate tissue-specific inflammation, organ dysfunction, and chronic autoimmune pathology.

While many steps in the development and progression of autoimmune diseases are becoming clearer, numerous mechanisms remain poorly understood. Ongoing and future research aims to elucidate how genetic variants predispose individuals to disease, how environmental exposures act as catalysts, and how immune dysregulation causes tissue damage. Understanding the immune phenotype, the functional state of the immune system in patients with autoimmune diseases, will be critical for identifying new diagnostic markers and designing targeted, mechanism-based therapies. Ultimately, integrating insights from genetics, immunology, and environmental health will enhance our ability to predict, prevent, and treat autoimmune disorders more effectively. We believe that our novel approach targeting the defective inhibition of IL-2R second messenger pathways in Tregs from patients with autoimmune diseases represents a highly innovative and promising strategy for potentially treating a wide range of autoimmune diseases. Tregs constitutively express the CD25 chain of the IL-2R. By restoring proper Treg function through targeted delivery of NAEi’s bound as protein drug conjugates to fusion proteins of IL-2 or anti-CD25 mAbs, we can re-establish immune tolerance without broad immunosuppression. In conclusion, our novel targeted drug delivery approach correcting the defective inhibition of IL-2R second messenger pathways in Tregs from patients with autoimmune diseases represents a highly innovative and promising strategy for potentially treating a wide range of autoimmune diseases. By restoring proper Treg function through targeted delivery of NAEi’s, we can re-establish immune tolerance without broad immunosuppression. This approach would offer an effective, safer, and broadly applicable “off the shelf” treatment option for patients with various autoimmune conditions, addressing a significant unmet medical need and potentially transforming the treatment landscape for these chronic, debilitating diseases.
